# Comparison of personal and shared frameshift neoantigen vaccines in a mouse mammary cancer model

**DOI:** 10.1186/s12865-020-00350-3

**Published:** 2020-05-05

**Authors:** Milene Peterson, Sierra Nicole Murphy, John Lainson, Jian Zhang, Luhui Shen, Chris W. Diehnelt, Stephen Albert Johnston

**Affiliations:** 1 Center for Innovations in Medicine, The Biodesign Institute, Arizona State University, Tempe, AZ 85287 USA; 2 Calviri, Inc, Tempe, AZ 85284 USA

**Keywords:** Personalized cancer vaccine, Shared-neoantigen vaccine, Frameshift peptides, Breast cancer, Mouse model, Immunotherapy

## Abstract

**Background:**

It is widely hoped that personal cancer vaccines will extend the number of patients benefiting from checkpoint and other immunotherapies. However, it is clear creating such vaccines will be challenging. It requires obtaining and sequencing tumor DNA/RNA, predicting potentially immunogenic neoepitopes and manufacturing a one-use vaccine. This process takes time and considerable cost. Importantly, most mutations will not produce an immunogenic peptide and many patient’s tumors do not contain enough DNA mutations to make a vaccine. We have discovered that frameshift peptides (FSP) created from errors in the production of RNA rather than from DNA mutations are potentially a rich source of neoantigens for cancer vaccines. These errors are predictable, enabling the production of a FSP microarray. Previously we found that these microarrays can identify both personal and shared neoantigens. Here, we compared the performance of personal cancer vaccines (PCVs) with that of a shared antigen vaccine, termed Frameshift Antigen Shared Therapeutic (FAST) vaccine, using the 4 T1 breast cancer model. Sera from 4 T1-tumor bearing mice were assayed on the peptide microarray containing 200 Fs neoantigens, for the PCV, the top 10 candidates were select and personal vaccines constructed and administrated to the respective mice. For the FAST, we selected the top 10 candidates with higher prevalence among all the mice challenged. Seven to 12 days challenged mice were immunized, combined or not with immune checkpoint inhibitor (ICI) (αPD-L1 and αCTLA-4). Primary and secondary tumor clearance and growth were evaluated as well as cellular and humoral immune response against the vaccine targets by IFN-γ ELISPOT and ELISA. Lastly, we analyzed the immune response of the FAST-vaccinated mice by flow cytometry in comparison to the control group.

**Results:**

We found that PCVs and FAST vaccines both reduced primary tumor incidence and growth as well as lung metastases when delivered as monotherapies or in combination with ICI. Additionally, the FAST vaccine induces a robust and effective T-cell response.

**Conclusions:**

These results suggest that FSPs produced from RNA-based errors are potent neoantigens that could enable production of off-the-shelf shared antigen vaccines for solid tumors with efficacy comparable to that of PCVs.

## Background

There has been rapid growth in development of vaccine approaches that direct the immune system to target tumor neoantigens for treatment [1]. The most successful approaches have identified patient specific neoantigens to produce personal cancer vaccines (PCVs) that are in multiple clinical trials [2–5]. Generation of PCVs relies on sequencing the DNA and RNA of the tumor to discover and validate mutations creating neoantigens. Algorithms or in vitro testing determine which mutations are likely to elicit an anti-tumor immune response and the vaccine is manufactured as peptides, DNA, RNA or viruses presenting the neoantigens [6]. The patient is then administered the unique vaccine, often in combination with another immunotherapeutic. Early reports on clinical trials applying such a system are encouraging in that the vaccines appear safe and may have signs of efficacy [2–5]. However, the PCV approach involves considerable time to produce the vaccine and may be costly. A pre-made vaccine would not suffer these limitations but may be less effective. Here we compare both types of vaccine in the mouse mammary tumor model.

Besides the time and cost issues, the biggest limitation of PCVs may be the dearth of DNA mutations in most tumors [6–8]. While some tumors have 100–1000s of non-synonymous single nucleotide variants (SNVs), a large number have few. For example, in a recent report on PCVs to melanoma, two out of 10 enrolled patients did not have the high mutation rates normally observed in melanoma, disqualifying them for vaccination [5]. The majority of other tumor types rarely have large numbers of mutations [7]. Since less than 5% of the mutations are expected to be immunogenic, this is a severe limitation on the breadth of application of the PCV strategy [8]. This has spurred exploration of other neoantigen sources focused on alternative splicing and from non-coding regions of the genome [8, 9].

In contrast to SNV neoantigens, we have found frameshift neoantigens (FS) produced by mistakes in RNA production and processing are a rich neoantigen source [10, 11]. Increasing evidence demonstrates that Fs neoantigens are efficacious, highly immunogenic antigens [12]. While rare in DNA, they are frequently produced by transcription through microsatellites in coding regions or by mis-splicing of exons, processes that are highly error-prone in tumors relative to normal cells [13–15]. We have shown that these RNA-based FS peptides (FSP) are protective in mouse tumor models and that these FSP elicit antibody and T-cell responses [10, 11]. Based on these findings we designed a small array of 788 human FSPs that were predicted informatically from exon mis-splicing or indels from transcription through microsatellites and used these arrays to screen 2 different murine cancers for personalized and shared immunoreactive FSP neoantigens. We observed that many FSP antigens were recurrent at various frequencies across individuals within a tumor type. We then designed a shared antigen, FAST vaccine for both tumor types as well as a PCV for each mouse in order to test the relative efficacy of these two FSP neoantigen vaccines in the 4 T1 metastatic breast cancer mouse model. This murine model is widely used to assess novel therapies against stage IV human breast cancer due its poor immunogenicity and ability to metastasize, shared characteristics of human advanced cancers [16, 17]. Metastatic breast cancer is the second most deadly cancer for US women, affecting approximately one in eight women in their lifetimes. With 41,000 deaths in 2018, current treatments and early detection have only decreased mortality rates by a modest 1.3% during 2011–2017 [18, 19]. Despite many advances in cancer research and improvements in patient care, a more effective treatment is still desperately needed.

## Results

### Development of PCVs and FAST vaccines

**Fig. 1:**
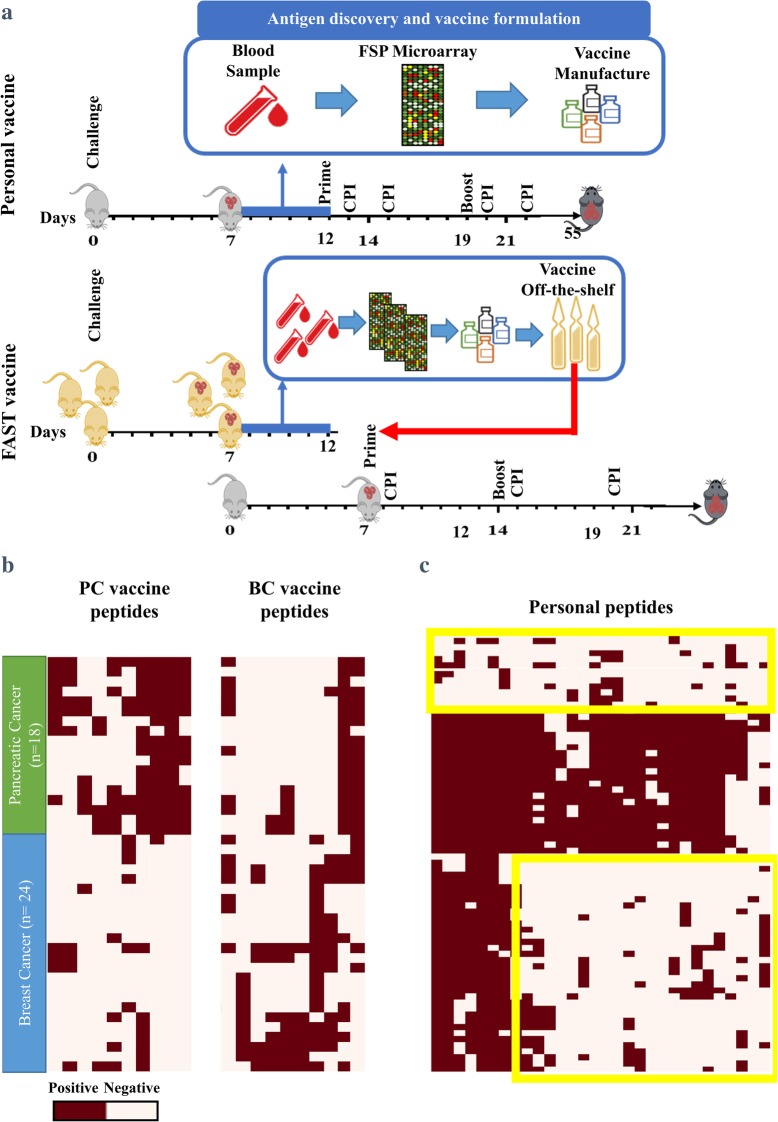
Frameshift (FS) PCV and FAST vaccine design and peptide selection. **a** Schematic diagram of PCV and FAST Fs vaccine approaches. Mice were subcutaneously (s.c.) inoculated with 4 T1 tumor cells and, seven days later, sera were collected. Pre- and post-challenge sera were assayed on FSP microarrays. For PCVs, the top 10 peptides with the highest median fluorescent intensity were selected for each mouse, individual PCVs constructed and administrated to the respective mouse (*n* = 10). A breast cancer FAST vaccine (BC-FAST) was composed of the top 10 candidates with the highest prevalence across all 4 T1 challenged mice (*n* = 24). Mice (n = 10/group) were vaccinated with PCVs s.c. (opposite side of the challenge) on days 12 and 19, or with FAST vaccines on days 7 and 14. For the ICI co-treated groups, anti-PD-L1 (200 μg/dose) and anti-CTLA-4 (100 μg/dose) were administrated intraperitoneally (i.p.) on days 13, 15, 20, 22 (PCVs) and 8,15 and 22 (FAST). **b** Heat map of the FS peptides selected for BC-FAST and pancreatic cancer FAST (PC-FAST) vaccines. **c** Heat map of the FS peptides selected for PCVs

In order to develop both the PCV and FAST vaccines we synthesized 788 peptides (20mers) representing 200 different Fs neoantigens. These peptides were spotted on glass slides and used to screen mouse sera. The basic procedure to make the vaccines and the challenge protocol is depicted in Fig. 1a. To produce PCVs, mice were injected with 500 4 T1 tumor cells and 7 days later, blood from each mouse was screened on the peptide microarrays for antigen discovery. For each mouse the 10 most reactive FSPs, relative to non-cancer controls, were chosen for the vaccine. As many FSPs were represented by more than 1 peptide on the microarray, each mouse received between 13 and 23 individual peptides (Fig. 1c, Supplementary Table 1).

While most FSP reactivities were personal to a single mouse, others were reactive in multiple mice, not surprisingly given that the mice are an inbred strain that received the same cell line. To develop the breast cancer FAST (BC-FAST) vaccine, the same data was analyzed and the 10 FS peptides that were the most frequently positive across all 24 samples were chosen as the shared antigens (Fig. 1b, Supplementary Table 2). The same process was applied using the mouse pancreatic cell line, KPC, to produce the PC-FAST vaccine. The frequency of occurrence of the reactive peptides chosen for 4 T1 and KPC is shown in Table 1 and Fig. 1b.

**Table 1:** FAST vaccines peptides selected and positive rates per cancer-type studied in mice model

Vaccine	Peptides	Positive rates (%)	
		Cancer Samples	
		Breast	Pancreatic
BC-FAST	CIMpep404	83.3	0
	CIMpep368	54.2	6
	CIMpep131	37.5	0
	CIMpep187	33.3	28
	CIMpep1	33.3	100
	CIMpep332	29.2	0
	CIMpep450	29.2	0
	CIMpep491	29.2	11
	CIMpep64	29.2	44
	CIMpep14	29.2	89
PC-FAST	CIMpep145	0	78
	MS-75	0	100
	CIMpep105	4.2	33
	CIMpep3	4.2	28
	MS-76	4.2	94
	CIMpep479	12.5	50
	CIMpep282	16.7	44
	CIMpep493	16.7	33
	CIMpep7	25	28
	CIMpep362	29.2	89

### BC-FAST and FS PCVs significantly reduce primary tumor growth as monotherapies

Each vaccine was tested against 4 T1 primary tumors according to the procedure outlined in Fig. 1a. BALB/c mice were injected with 500 4 T1 cells and were treated with either BC-PCV on day 12 and 19 or BC-FAST on day 7 and 14. We chose to vaccinate the FAST vaccine 5 days earlier than the PCV to reflect the advantage of a premade vaccine over a personal one. Human PCVs generally take months to produce. The low injection number of 4 T1 cells was required to have sufficient time to prepare the PCV, simulating the time lag to produce PCVs for human use. Each vaccine was administered with poly (I:C) as adjuvant. The control set (Mock) consisted of mice injected with PBS. As another negative control, a non-reactive vaccine (NR) was prepared from FS peptides that were not reactive with sera from 4 T1 tumor bearing mice (Supplementary Fig. 1). An additional cohort of mice were vaccinated with the PC-FAST vaccine on the same schedule as the BC-FAST vaccine.

**Fig. 2:**
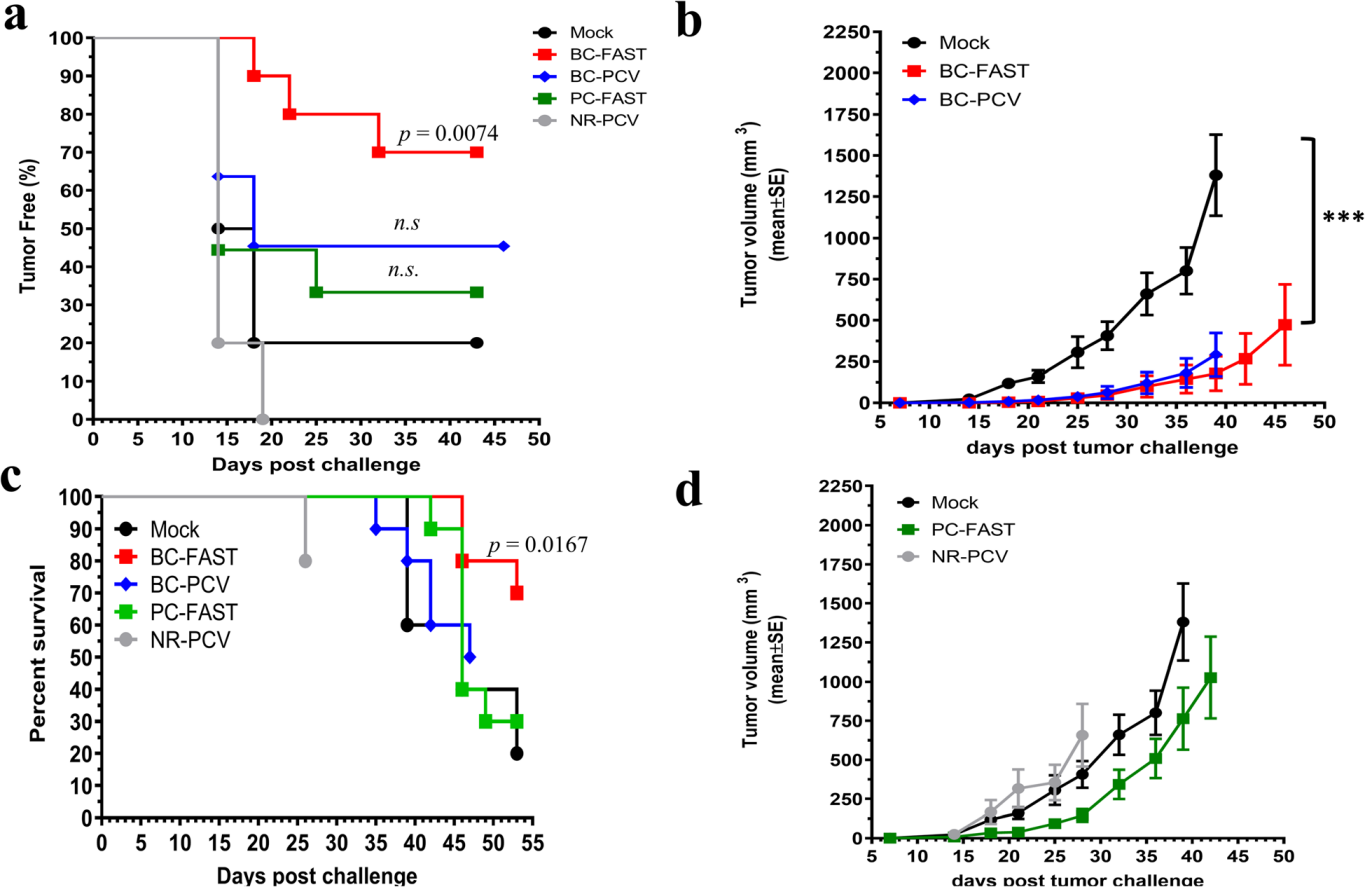
BC-FAST and PCV treated mice control primary tumor growth. **a** Percentage of tumor free mice. Female Balb/c mice (n = 10/group) were challenged, vaccinated and tumor volume was measured twice per week. Mice were vaccinated with PBS (Mock), BC-PCV, BC-FAST, PC-FAST, or NR-PCV. Data are presented as percentage of tumor free mice and *p* values calculated using Log-rank (Mantel-Cox) Test. **b** Average 4 T1 tumor growth curves. The tumor volume of 4T1-tumor bearing mice was measured and plotted for BC-PCV, BC-FAST, or Mock vaccinated mice (b) and for PC-FAST and NR-PCV vaccinated mice (d). **c** Kaplan-Meyer survival curves of vaccinated mice. At 47–55 days post challenge, mice with tumor volume < 1000 mm^3^ and/or no clinical signs of illness (weight loss, skin ulceration, hair loss or lethargy) were considered survivors. Data were analyzed using Log-rank (Mantel-Cox) Test (*** *p* < 0.001)

Tumor growth was monitored for each group. Seven out 10 BC-FAST vaccinated mice were tumor free versus 2 out of 10 mock vaccinated mice (*p* = 0.0074) (Fig. 2a, Table 2). There were 5 tumor-free mice in the BC-PCV vaccinated group (n.s) while the PC-FAST vaccinated group had similar tumor-free curves (3/10) as the control arm. All mice vaccinated with NR developed tumors. For the tumor-bearing mice, the PCV and BC-FAST vaccines significantly delayed tumor growth and reduced the tumor volume at the endpoint (Fig. 2b, Supplementary Fig. 2). However, in contrast to the BC-FAST and PCV vaccinees, the tumors in the NR and PC-FAST groups grew as fast as the control group (Fig. 2d). Additionally, BC-FAST vaccination prolonged survival in comparison to all the other vaccine groups and mock (*p* = 0.00167)(Fig. 2c).

**Table 2:** Number of tumor-free mice per vaccine group after challenge and re-challenge

Vaccine group	Tumor-free mice after	
	1st challenge	2nd challenge
Mock	2/10	0/2
BC-FAST	7/10	1/7
BC-PCV	5/10	N/T
PC-FAST	3/10	0/3
NR-PCV	5/5	N/T
BC-FAST/ICI	4/10	1/4
BC-PCV/ICI	3/10	N/T
PC-FAST/ICI	4/10	0/4
NR-PCV/CI	2/10	N/T

*N/T* not tested

Tumor-free mice in the mock, BC-FAST and PC-FAST groups were re-challenged with twice as many 4 T1 (1,000) cells on day 43 after the initial inoculation (Supplementary Fig. 3, 5) and received a vaccine boost 21 days post re-challenge (day 64). Although a majority of mice developed tumors, tumors in the BC-FAST group grew at a slower rate than the controls (*p* < 0.01) while the delayed tumor growth of PC-FAST vaccinated mice was not statistically significant. Intriguingly, 2 mice vaccinated with BC-FAST, with and without ICI, had a small nodule that remained the same size during the entire re-challenge experiment (35 days). Remarkably, BC-FAST vaccinated mice that received prior ICI treatment (BC-FAST/ICI) when re-challenged showed an increased tumor latency (24 days versus 19 days) and had even smaller tumors (average 307 mm^3^) than control (average 1100 mm^3^) and vaccine alone (average 607 mm^3^) (Supplementary Fig. 3), suggesting a long-term immune-stimulation effect by the combined immunotherapy [20]. These results indicate that the BC-FAST vaccine may be as effective as the BC-PCV in this model.

### PCVs and FAST vaccines in combination with ICIs

**Fig. 3:**
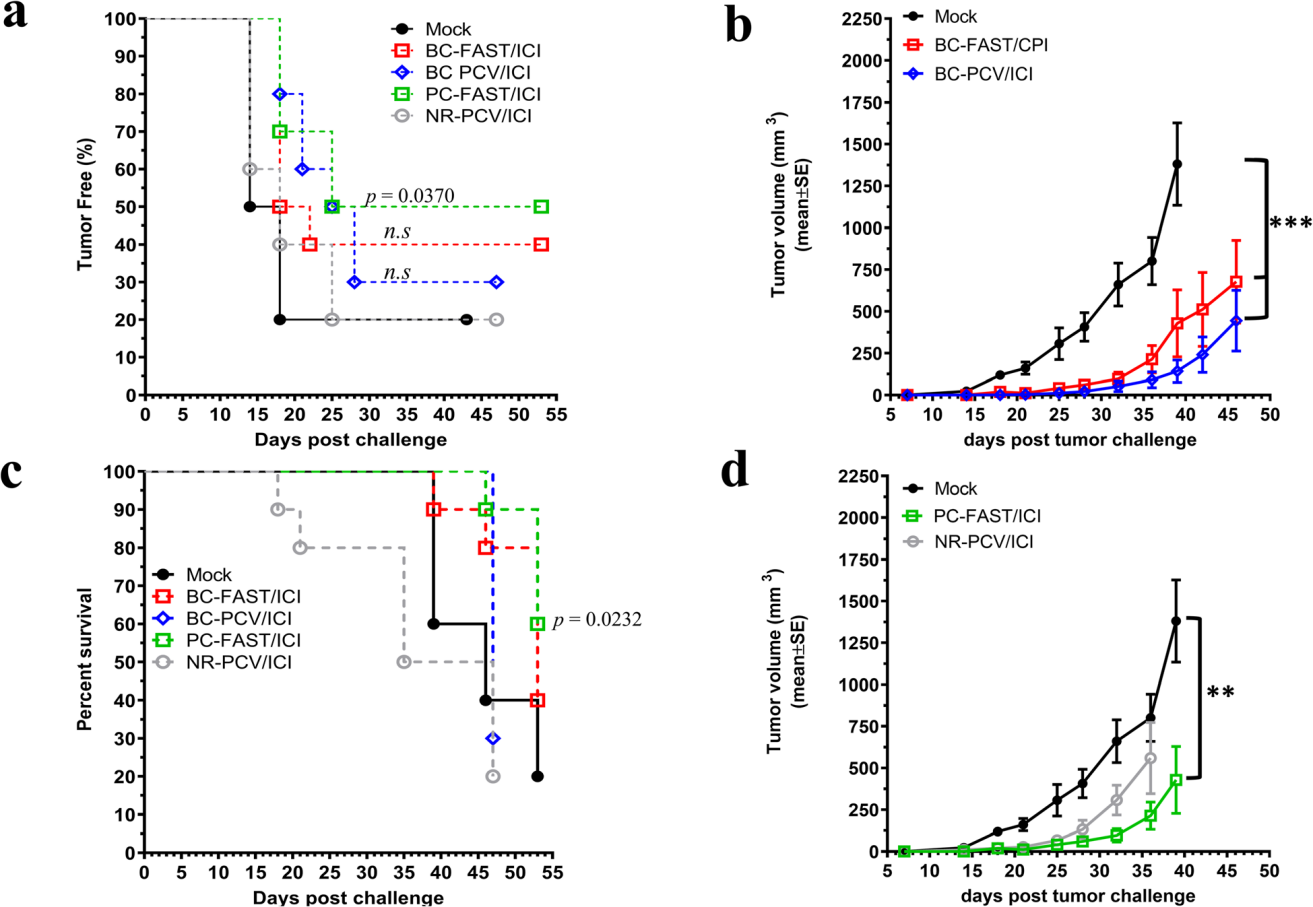
Fs vaccines combined with immune checkpoint inhibitors reduced tumor growth and incidence. **a** Percentage of tumor free mice. Mice were challenged, vaccinated and treated with checkpoint inhibitors (anti-PD-L1/ CTLA-4) as described in Fig. 1a. Mice were monitored for tumor incidence and tumor volume. Data are presented as percentage of tumor free mice and *p* values calculated using Log-rank (Mantel-Cox) Test. **b** Average 4 T1 tumor growth curves. Data were analyzed by Log-rank (Mantel-Cox) Test (***, p < 0.001). **c** Kaplan-Meyer survival curves of vaccinated mice. At 47–55 days post challenge, mice with tumor volume < 1000 mm^3^ and/or no clinical signs of illness were considered survivors. Data were analyzed using Log-rank (Mantel-Cox) Test. **d** Average 4 T1 tumor growth curves. The tumor volume of tumor bearing mice was measured and plotted for PC-FAST/ICI and NR-PCV/ICI, or Mock vaccinated mice. Data were analyzed by Log-rank (Mantel-Cox) Test (**, *p* < 0.01)

As many current clinical trials of PCVs include ICI treatment, we tested the effect of anti-PD-1 plus anti-CTLA-4 ICI treatment on FAST and PCV efficacy. In these studies, PCV treated mice received 4 ICI injections after the peptide vaccinations while FAST vaccinated mice received 3 ICI injections. We reduced the number of ICI doses due to the rapid and fatal reactions in controls groups after repeated administration of the ICI (4–5 doses) observed in our study and, previously observed and reported by Mall et al. 2016 [21]. Interestingly, while the BC-FAST/ICI and BC-PCV/ICI groups did not show statistically significantly improved tumor-free curves relative to the vaccine alone or the Mock group, the PC-FAST vaccine did (*p* = 0.0370) (Fig. 3a). In fact, we observed an earlier tumor onset in the presence of ICI for BC-FAST group, probably due to the immune cell infiltration causing the phenomenon called “pseudoprogression” [22, 23]. Similar to the monotherapy results, those mice that developed tumors had significantly slower growth in the PC-FAST/ICI, BC-FAST/ICI and PCV/ICI groups relative to controls (Fig. 3b). However, the BC-FAST/ICI group showed slightly larger tumors than the vaccine alone, indicating again possible pseudoprogression. Importantly, the addition of ICI to the BC-PCV group extended their survival window by 10 days. Morbidity for NR-PCV/ICI vaccinees exceeded that of the control mice and this group had to be sacrificed. As observed for the vaccine therapy alone, the number of tumor free mice and survival rates were improved compared to the control group (Fig. 3a, c and Table 2). We conclude that including ICI did not improve the BC-FAST vaccine performance, but did potentiate the BC-PCV and pancreatic cancer (PC-FAST) vaccines efficacies.

### PCVs and FAST vaccines reduce lung metastases

**Fig. 4:**
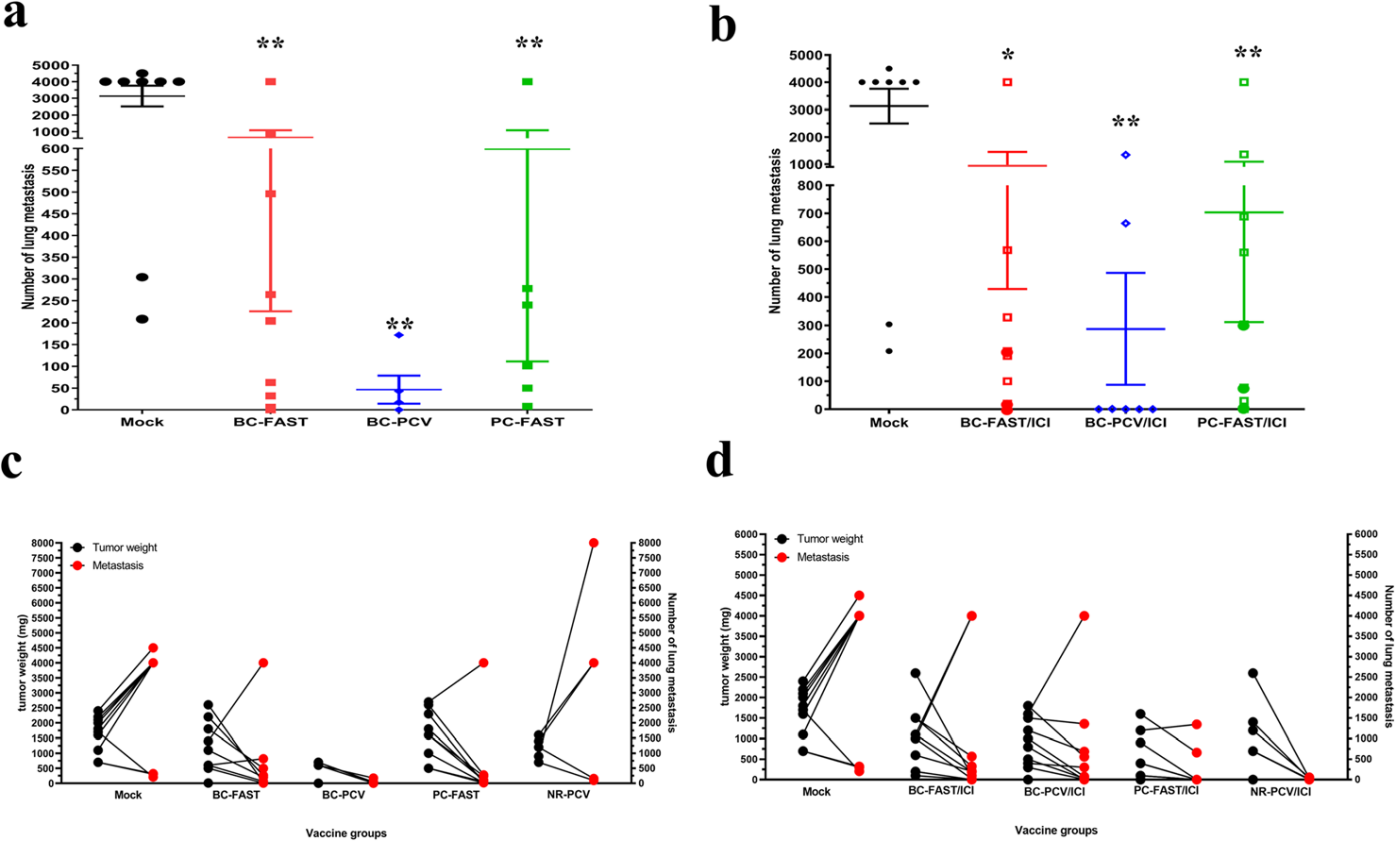
Fs vaccines reduce spontaneous lung metastasis. **a** Quantification of pulmonary metastasis after immunization with Fs vaccines alone or (**b**) in combination with checkpoint blockade. At the endpoint, mice were euthanized, the lung aseptically removed, enzymatically treated and plated for 14 days. Fixed clonogenic 4 T1 metastatic colonies were stained with methylene blue and then counted. Bar graphs represent mean ± S.E and dots represent counts for each mouse evaluated. * p < 0.01; **, *p* < 0.001, analyzed by unpaired t test with Welch’s correction. Circle symbols in both vaccines represent re-challenged mice. Correlation between tumor weight (at endpoint) and number of pulmonary metastasis without (**c**) and with ICI treatment (**d**). Dots represent each mouse evaluated

The effect on the incidence of secondary spontaneous tumors was also assessed. We noted significant reductions in lung metastases for the BC-FAST vaccination (with and without re-challenge (circles)), BC-PCV and PC-FAST (with and without re-challenge) vaccination versus the control mice (Fig. 4a). The average number of metastases observed in the PCV group was less than the FAST vaccinated groups, though this result was non-significant. Additionally, vaccinated tumor-free mice did not have measurable lung metastases. Similar to the monotherapy results, there was a significant reduction in lung metastases for the BC-FAST/ICI, PCV/ICI and PC-FAST/ICI groups versus control but non-significant differences between groups (Fig. 4b). Surprisingly, association between tumor size (weight) and the number of pulmonary metastases revealed an enhanced efficacy of both, the tumor specific (BC-FAST) and non-specific (PC-FAST), FAST vaccines in controlling of tumor dissemination (Fig. 4c). While mock and negative control (NR-PCV) groups demonstrated that larger primaries tumors also have larger number of metastases, FAST vaccinated animals with large tumors had fewer pulmonary tumors. This was not observed in mice vaccinated with PCV as monotherapy. By adding ICI to the PCVs or NR vaccine (negative control), produced a similar pattern observed for the FAST vaccines, but did not improved FAST anti-metastasis capacity (Fig. 4d). Altogether, these data suggest that the anti-metastatic mechanism of the PCV and FAST, as monotherapies, are different; while PCV reduces tumor dissemination by controlling primary tumor, FAST vaccines control pulmonary metastases regardless its efficacy against the primary tumors.

### BC-FAST and PCVs elicit robust neoantigen specific T-cell responses

Peptides for the PCVs and the BC-FAST vaccines were selected based upon antibody reactivity on the microarrays, so we expected them to also be reactive by ELISA. The antibody response from pooled sera (collected at the endpoint) to each peptide in the BC-FAST vaccine demonstrated that, as expected, the injection of the tumor alone in the Mock mice elicited an antibody response to most peptides (Supplementary Fig. 6). Interestingly, vaccination with BC-FAST or PC- FAST alone or with ICI treatment did not generally increase the antibody responses (Supplementary Fig. 6). For the PCV group, each FS antigen was assessed in each mouse and it was found that most peptides were reactive to varying degrees in each mouse (Supplementary Fig. 6).

**Fig. 5:**
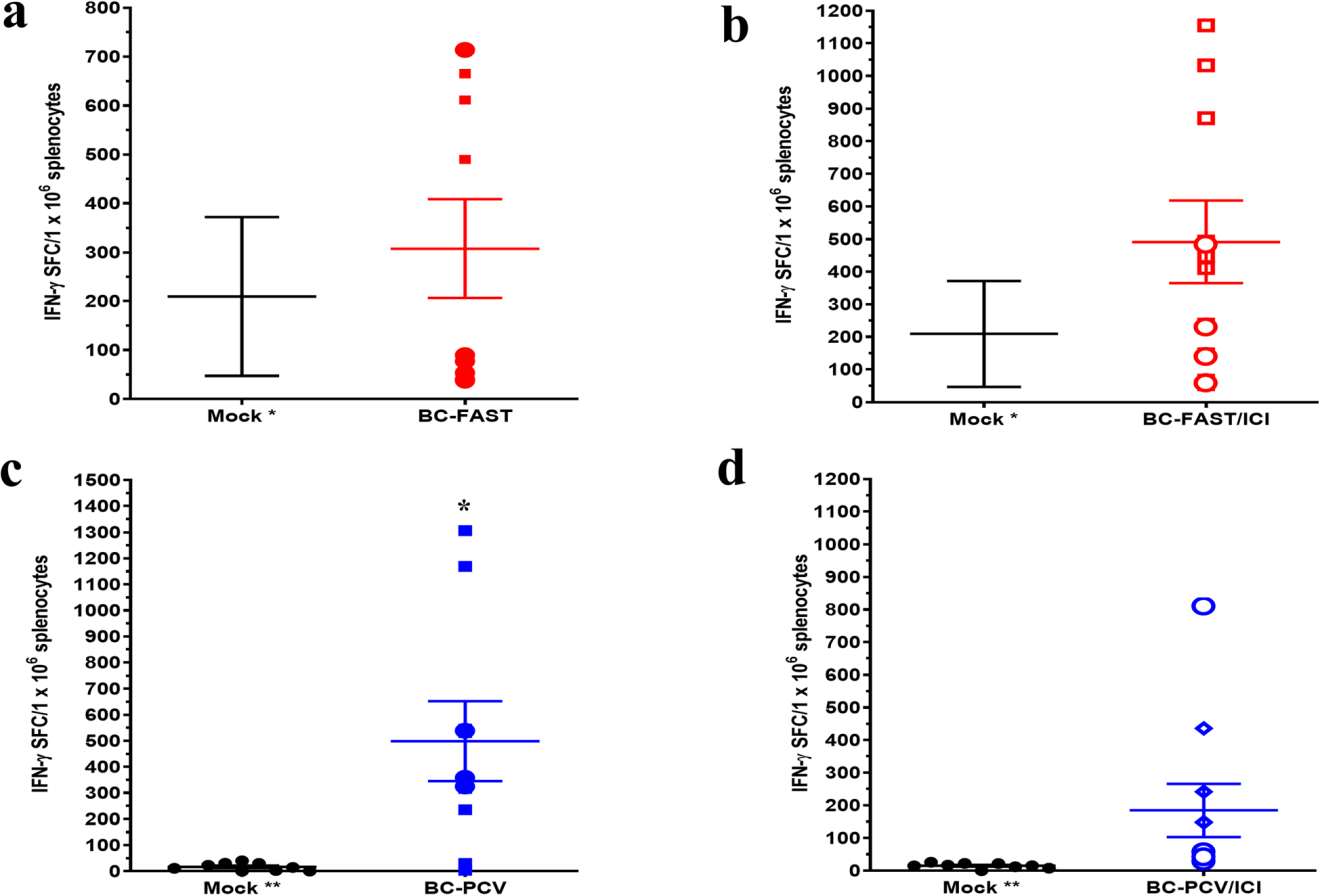
FS vaccination induces robust T-cell responses. IFN-γ positive splenocytes against shared BC-FAST antigens without (**a**) and with ICI (**b**). At the endpoint, mice splenocytes were harvested, stimulated in triplicate with pooled vaccine peptides (10 mg/ml per well of each peptide) (3–4 Fs antigen/well) and assessed by IFN-γ ELISPOT. Mouse splenocytes from the mock group were pooled and evaluated against all the peptide pools for BC-FAST. And against each of the PCV formulations (all vaccine antigens together) without (**c**) and with ICI (**d**). Bars represent mean ± S.E for the vaccine groups and dots represent per mouse immune response to the vaccine formulation (sum of the vaccine peptide pools). The Mock group was tested twice, and bars represent the mean ± S.E between replicates. (Circles) Re-challenged mice. * *p* < 0.05, analyzed by unpaired t test with Welch’s correction. Mock* = tested against BC-FAST vaccine peptides; MOCK** = tested against PCVs peptides

**Fig. 6:**
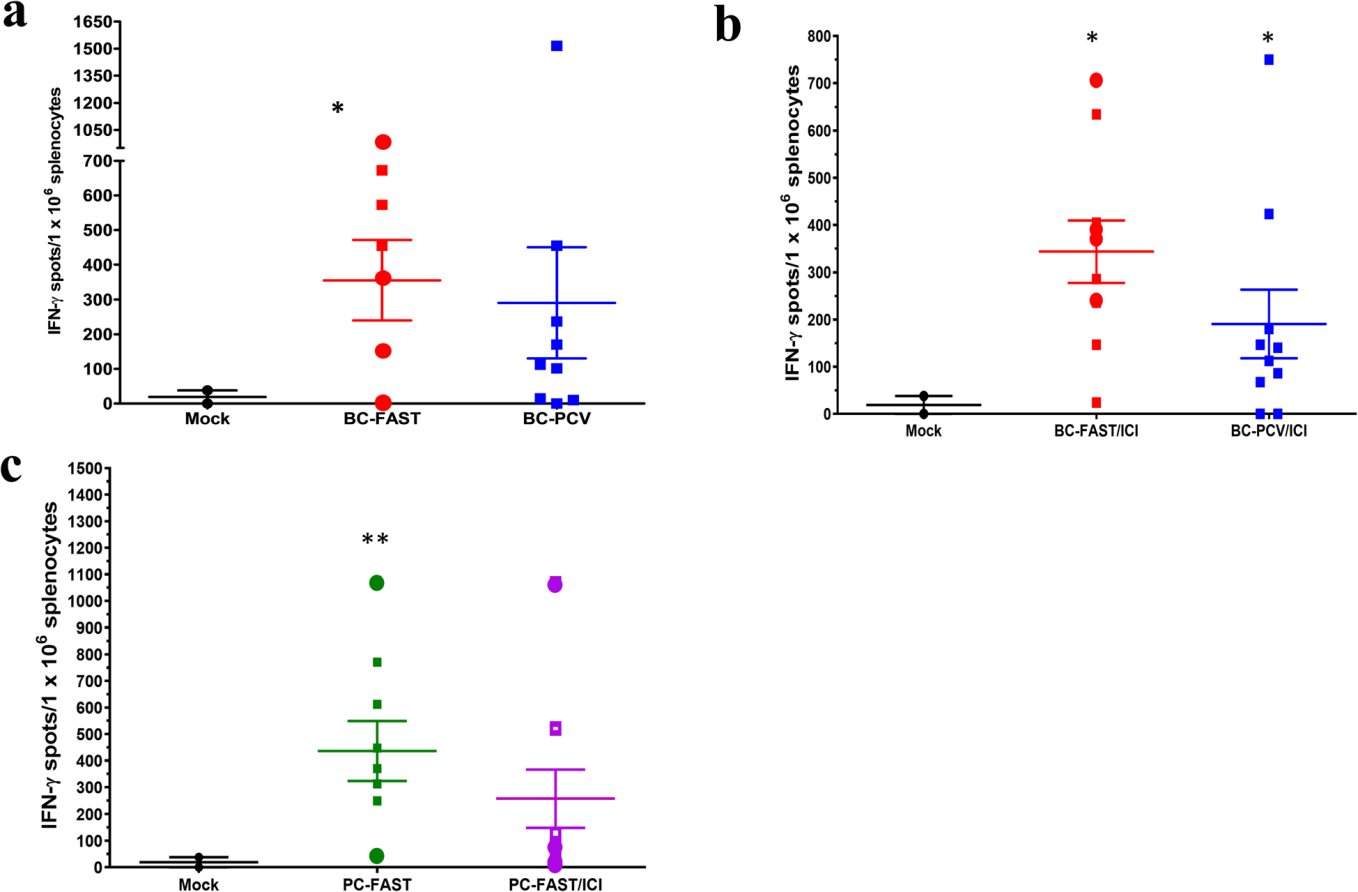
FS vaccinated mice have T-cells that recognize 4 T1 tumor cells. IFN-γ positive splenocytes stimulated by 4 T1 cells from (**a)** BC-FAST or BC-PCV treated mice, Mouse splenocytes were stimulated, in triplicate, with 4 T1 cells (1 × 10^5^ cells/well) for 72 h and evaluated by IFN-γ ELISPOT. IFN-γ positive splenocytes stimulated by 4 T1 cells from **a** BC-FAST or BC-PCV mice (**a**) without and (**b)** with ICI, or (**c)** PC-FAST treated mice with or without ICI treatment. Bars represent mean ± S.E for each group and dot represents mouse individual immune response. Pooled mock splenocytes were tested in two independent experiments, and dots represent each replicate and bars mean ± S.E of these experiments. * *p* < 0.05; **, *p* < 0.001, analyzed by unpaired t test with Welch’s correction

We also evaluated the T-cell responses to the FS antigens and found that the Mock vaccinated group had a moderate T-cell response to the shared antigens in the BC-FAST vaccine but did not react with the PCV antigen peptides, as measured by ELISpot of mouse splenocytes (Fig. 5a,c). However, vaccination with the BC-FAST (n.s.) vaccine or PCVs (*p* = 0.01) did produce a T-cell response to pooled peptides in most of the mice (Fig. 5a,c, Supplementary Fig. 7). However, addition of ICI to either group did not significantly affect the number of reactive T-cells (Fig. 5b,d). The T-cell response against the 4 T1 tumor cells was measured and as expected, there was little reactivity in the Mock group. However, the BC-FAST, BC-PCV and PC-FAST vaccinees had significant T-cell responses against the tumor in most of the vaccinated mice (Fig. 6).

### BC-FAST evokes a robust cellular immune response

**Fig. 7:**
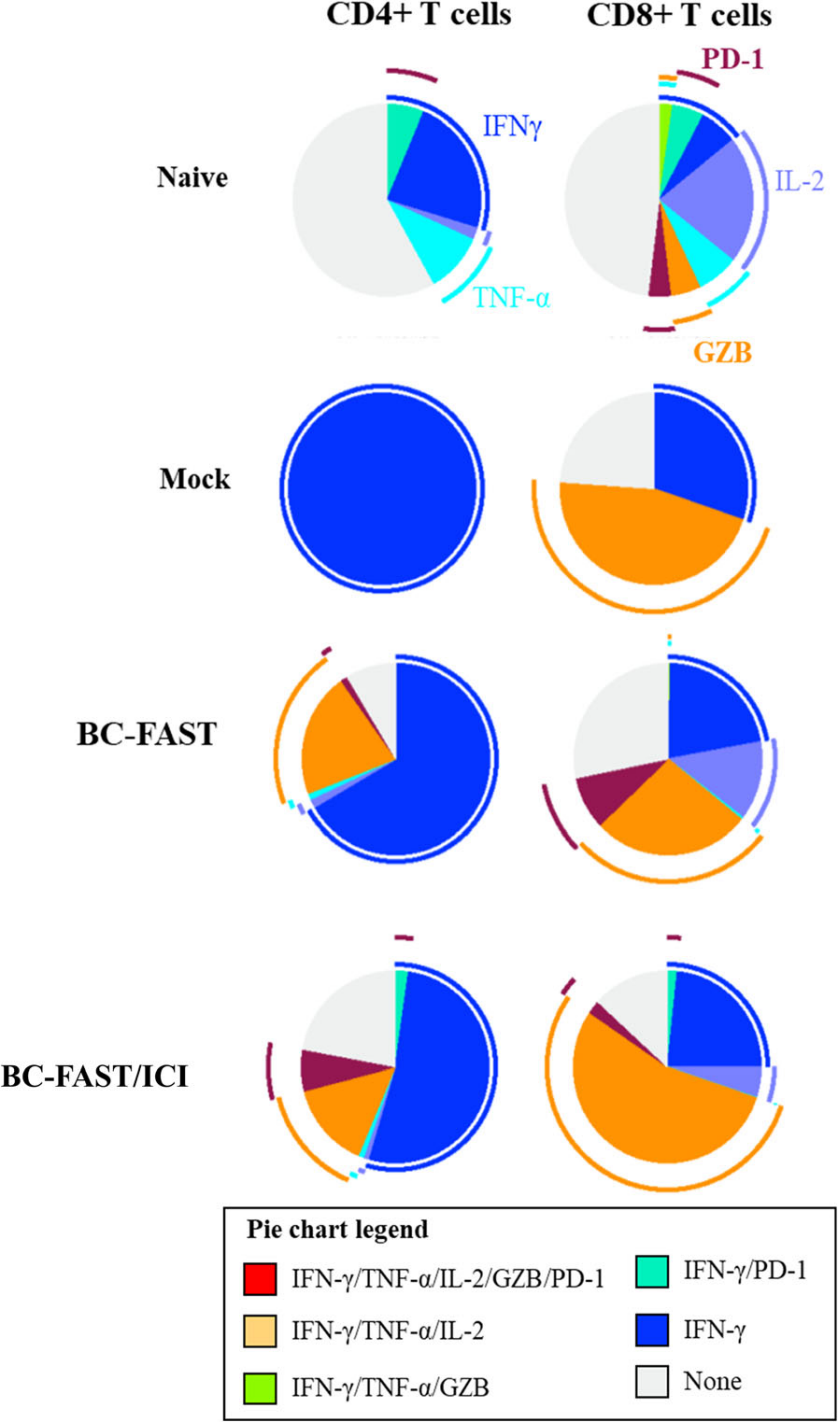
BC-FAST vaccinated mice have functional CD4+ and CD8+ T cells. Multiparameter flow cytometry was used to determine the percentages of the cytokine generation in both CD8+ and CD4+ T cell of mice immunized with BC-FAST vaccine alone or in combination with ICIs. Naïve mice and Mock vaccinated groups are indicated at the top. Pie charts show the relative proportion for each tested cytokine population as well the Boolean combination gates for IFN-γ +, TNF-α +, IL-2+, GZB + and PD-1+. Arc charts shows the production of each individual cytokine/ surface marker. Assays were performed with cryopreserved cells without any prior in vitro expansion. Data is representative of average of cytokine production percentage from: Naïve = 3 mice; Mock, BC-FAST and BC-FAST + ICI = 5 mice each group

To better understand the phenotype of the T-cell populations produced by the BC-FAST vaccine, we used FACS to evaluate the cytokine-producing T cell populations in naïve BALB/c mice and vaccinated tumor-bearing mice with splenocytes stimulated in vitro by BC-FAST peptides (Fig. 7). In naïve mice, we observed the presence of CD4+ T-cells producing IFN-γ, TNF-α, or IL-2 cytokine in response to ex vivo stimulation by the shared FS peptides. This indicates a Th1 subtype profile, but no polyfunctional T-cells were observed. Naïve CD8+ T cells showed a high frequency of single-positive cells for cytokines (IFN-γ, TNF-α or IL-2) and granzyme B, with low frequency of triple-positive effector cells (IFN-γ, TNF-α and Granzyme B). Interestingly, we observed a small frequency of IFN-γ and PD-1 double positive cells in both CD4+ and CD8+ T-cells. PD-1 expression in effector T cells has a complex significance, suggesting both T cell dysfunction as well as T cell activation/high avidity [24]. These data indicate the immunogenicity of these peptides and their ability to induce antigen-specific effector cells.

Mock vaccinated mice had a predominantly Th1 CD4+ T-cell response with production of IFN- γ and CD8+ T-cells that were positive for IFN-γ or granzyme B. In contrast, BC-FAST vaccinated mice, both with and without ICI treatment, had T-cells that could produce effector cytokines (IFN-γ, TNF-α or IL-2) and degranulate (Granzyme B). Furthermore, we detected an increased frequency of CD4+ T cells producing granzyme B indicating their cytotoxic activity (CTL), possibly as a result of chronic exposure to the tumor [25]. Similar to naïve mice, we detected an increase in the frequency of cells single and double-positive (with IFN-γ) for PD-1, indicating a exhaustion/activation phenotype. Altogether, these data demonstrate that FAST vaccination induces T-cells with potent ability to produce both effector cytokines and degranulate, despite PD-1 expression.

## Discussion

In this study, we designed PCVs and an off-the-shelf, shared neoantigen vaccine (BC-FAST) composed of frameshift neoantigens against a triple-negative breast cancer cell line (4 T1) and compared their efficacy and immune responses. A peptide microarray composed of FS neoantigens predicted to be generated by errors in RNA processing, either from exon mis-splicing or transcription through INDELs in microsatellite regions, was used to identify neoantigens for both vaccine types. When used as monotherapies, both vaccines conferred comparable protection to primary tumor growth in a therapeutic model and both reduced lung metastases relative to a Mock vaccine control. Importantly, a shared antigen vaccine designed against a pancreatic tumor cell line (PC-FAST) and a vaccine composed of non-reactive (NR) peptides from the array did not reduce primary tumor growth. ICI co-treatment did not enhance protection to the primary tumor nor did it reduce metastasis for PCVs or BC-FAST vaccinated mice relative to vaccine alone. All vaccines elicited strong T-cell immune responses to their component antigens and to tumor cells, with variable antibody responses to the antigens.

The primary goal of this study was to test the relative efficacy of PCVs and shared frameshift antigen vaccines in a breast cancer model. This comparison was possible due to the predictable nature of FS antigens from RNA processing errors. This predictability enabled pre-synthesis of a small library of possible candidate antigens that could be screened against patient sera to discover immunogenic antigens that are either specific to the patient or shared across the group [11]. This made it possible to quickly identify antigens for rapid PCV formulation for each mouse. This approach would not be feasible for neoantigens identified from DNA mutations as sequencing and vaccine synthesis could not be completed before mice succumbed to the tumors. Since there are only ~ 200 K possible FSP from exon mis-splicing, we were able to previously screened microarrays containing all possible FS against an number of cancer types which allowed us to choose 200 peptides to be synthesized for this effort [11]. This system enabled PCV identification and vaccine production for each mouse in less than 5 days, as opposed to a month or more if from DNA mutations. Choosing commonly reactive peptides enabled BC-FAST vaccine production before challenge. For development of FAST vaccines for clinical application the full 200 K peptide arrays would be used to select the best peptides for production of a standard, off-the-shelf vaccine.

In humans the optimal application of the personal cancer vaccine approach would be that at diagnosis of cancer the process of production of the PCV would initiate. After sequencing of the tumor, assuming the patient had enough mutations to be eligible, the process would take at least 1–3 months before administration of the vaccine [2–4]. During this time the tumor could evolve and advance. In contrast, with the FAST vaccine all patients could be administered the vaccine on diagnosis. The protocol we used to compare the FAST vaccine and PCV in mice was designed to reflect this difference in clinical protocols. The FAST vaccine, because it was pre-made, was administered on day 7. For the PCV the process of producing the vaccine was initiated on day 7 and the vaccine delivered on day 12, the fastest we could design and produce the vaccine.

These results demonstrate several possible advantages of FS neoantigens from RNA processing errors for cancer vaccines. While it is possible to discover PCV neoantigens with this approach, the more interesting aspect is the relative high occurrence of shared neoantigens across mice and in patient populations [10, 11], in contrast to neoantigens from DNA mutations. This could enable development of off-the-shelf vaccines for specific cancers overcoming the logistical challenges of PCV discovery and manufacturing that cost patients precious time to treat. It is also noteworthy that these neoantigens were readily identifiable from a “cold tumor” with low mutational load [7] suggesting it could be possible to develop FAST vaccines against other cold tumors.

Frameshift peptide microarrays as an antigen discovery technology, while an indirect measure of the presence of neoantigen in the tumor, are a direct measure of the immunogenicity of the neoantigens in the patient. This is in contrast to PCV approaches which identify the DNA-based neoantigen and then predict immunogenicity with mixed success at best [2–5]. It should be noted that neoantigens can evolve over time as was recently noted in colorectal cancers [26] and in pancreatic cancers which have different FSP neoantigen profiles in early stage and late stage [10, 11]. The dynamic neoantigen profile could adversely affect PCVs while an off-the-shelf shared vaccine should not be as affected by this process.

In this study, PCVs and FAST vaccines were discovered from a small library of 200 FSPs. We have shown with peptide microarrays of all 220,000 possible FSP, that ~ 20% of all peptides show reactivity for any particular cancer with many public antigens that are shared across 10% to up to 70% of patients [10, 11]. The shared FSP are not strictly tumor type specific and the amount of overlap between tumor types varies considerably, similar to the results of this study. The peptides chosen for the 4 T1 BC-FAST vaccine were shared between 29 and 83% of the 24 mice used. This was also true for the PC-FAST vaccine for the pancreatic cell line. As we had seen for the human samples, some FS peptides were reactive in both the 4 T1 (breast) and KPC (pancreatic) cell lines, which could explain the partial protection of the PC-FAST vaccine in the 4 T1 mouse model. In contrast to the BC-FAST vaccine which was potent as a monotherapy, the PC-FAST vaccine required combination with ICIs to protect against the primary tumor. This could be due to the PC-FAST vaccine containing only five 4 T1 reactive antigens versus the 10 antigens contained in the BC-FAST vaccine. This implies that the small number of 4 T1 antigens in the PC-FAST are not enough to limit the primary tumor but can reduce metastases and are more effective in combination with ICIs.

Immune analysis of FSPs selected for vaccination revealed that all groups had broad antibody responses to FS neoantigens after tumor challenge, consistent with the antigen discovery by FSP microarrays. It is interesting that the vaccines did not boost the antibody response but this is consistent with reports that the poly (I:C) adjuvant plus peptides preferentially enhances cellular immune responses [27, 28]. Importantly, antibody levels did not correlate with tumor size, as demonstrate by groups with larger tumors did not have higher antibody responses to all peptides tested. In contrast to the antibody response, T-cell analysis revealed a weak pre-existing T-cell reactivity to the FSPs in the PCVs, BC-FAST, or PC-FAST vaccines. However, vaccination induced a strong T-cell response whether T-cells were stimulated by peptides or the 4 T1 cells themselves. Others have also observed that most neoantigens chosen for vaccines have low or no pre-existing T-cell responses [2–5, 29]. While more mice remained tumor free in the BC-FAST and PCV vaccinated groups than in the controls, these tumor free mice grew tumors when re-challenged with twice the 4 T1 dose of cells. These secondary tumors did grow significantly slower in the BC-FAST group compared to the other re-challenged mice. One interpretation of these results is the BC-FAST induced enough T-cell response to kill or contain the initial dose of 4 T1 cells in most mice. However, that level of immune response could only slow tumor growth in the re-challenge with more tumor cells. For both FAST and PCVs it will remain a challenge to create enough effective T-cells to kill all the tumor cells.

Additionally, the shared frameshift peptides selected for the BC-FAST induced polyfunctional antigen-specific T cells, both CD4+ and CD8+, in naïve mice, indicating the capability of this peptide vaccine to elicited a protective and functional immune responses. Also, the pre-existing anti-FSP T cell immunity in tumor bearing mice (mock) presented a dysfunctional phenotype. On the other hand, the vaccination boosted FS antigen-specific effector T CD4+ and CD8+ cells, important for an effective antitumor immune response [30]. A sustainable and optimal effector antitumor response has been associated to a polyfunctional phenotype cells [31]. Most of our vaccine-induced T cells were monofunctional, suggesting repeated vaccination may be required to maintain a protective immune response.

We conducted these studies to determine if there may be value in pursing the FAST vaccine concept. The model we used has limitations relative to the clinical situation. We used the immune reaction to cell lines to define the FAST vaccines. In patients the variation in tumors will be much more than in the cell lines. A FAST vaccine for breast cancer would have to be defined across widely variable tumor types. It will be of interest to test different FAST vaccines against different mouse mammary tumor lines. Recently, we showed that for 5 different human cancers, it may be possible to define a FAST vaccine for each tumor type [11]. We also used inbred mice, where in humans the immune response to the vaccine will be much more variable, as has already been evident in personal cancer vaccine trials [2–5]. The personal vaccines were developed from RNA sourced FSPs, not DNA sourced neoantigens. Considering the potential advantages of FAST vaccines, the pre-clinical data presented here justify their further investigation.

## Conclusion

In conclusion, we demonstrate the feasibility of designing and producing both personalized and shared-neoantigen vaccines targeting RNA-based frameshift peptides and the therapeutic efficacy of these approaches against a poorly immunogenic and challenging preclinical model. Additionally, we validated the use of our frameshift peptide microarray to screen and select potential candidates for the anticancer vaccines, lowering the process interval and expenses required, mainly, for personalized immunotherapies. In our comparison the FAST vaccine and PCV had similar performance. Given the advantages in simplicity and cost of the FAST approach, we think our studies support further exploration of this type of vaccine.

## Methods

### Mice and tumor cell line

All mouse procedures and protocols were approved by the Arizona State University Institutional Animal Care and Use Committee (protocol #1568R). Animals were purchased from The Jackson Laboratory and housed at ASU specific pathogen free (SPF) at the Interdisciplinary Science and Technology Building 1 (ISTB1) administrated by the Department of Animal Care and Technologies (DACT). Mice were caged in a ventilated Thoren with 250 cages or less, room temperature of 74 ± 2 °F (23 ± 1 °C), light cycle of 12 h of light /12 h of dark and 10–15 air changes per hour. The 4 T1 cells were purchased from ATCC (ATCC® CRL-2539™) in 2006, cultured as specified and aliquots stored at − 180 °C until use. For the experiments, cells were used with no more than 5 passages in vitro and authentication was only made by the supplier (ATCC). Serum samples from the KPC pancreatic tumor mouse model were obtained from C57/BL6 mice challenged by Dr. Haiyong Han’s team, at TGen.

### 4 T1 BALB/c breast cancer mouse model and vaccination regimen

4 T1 tumor cells were grown in RPMI-1640 culture medium supplemented with 10 units/ml penicillin-streptomycin (Sigma Aldrich) and 10% fetal bovine serum (FBS) at 37 °C in 5% CO_2_ until 80% confluence. Cells were detached with trypsin (TrypLE, Thermo fisher Scientific), washed 3 times and suspended in sterile PBS 1X at 5 × 10^3^ cells/ml. 4 T1 cell suspension (500 cells in 100 μl PBS 1X) was inoculated subcutaneously (s.c.) into the right flank, near the mammary glands, of each female BALB/c mice (6–8 weeks-old) [19, 32]. Mice were randomized into the treatment groups (*n* = 10 mice/group) (5 mice/cage). Tumors were measured twice per week. For the FAST vaccine experiments, 4 T1-tumor bearing mice were immunized at day 7 and boosted at day 14. For the PCV experiments, 4 T1-tumor bearing mice were immunized at day 12 and boosted at day 19. Both vaccines were administrated subcutaneously in the left flank (opposite to the tumor injection). Each immunization was composed of 50 μg vaccine peptide pool (5 μg/FS peptide) and 50 μg Poly (I:C) (Polyinosinic–polycytidylic acid potassium salt, Sigma Aldrich) in 100 μl sterile PBS 1X. PCV peptides were conjugated to KLH according to the manufacturer’s protocol (Imject Maleimide-Activated mcKLH™, Thermo Scientific) and purified by dialysis. For the immunotherapeutic treatment, mice were treated with anti-mouse PD-L1 (200 μg/mouse, clone: 10F.9G2, BioXCell, West Lebanon, NH) and anti-mouse CTLA-4 (100 μg per mouse, clone: UC10-4F10–11, BioXCell, West Lebanon, NH) on days: 8, 15 and 22 (for the FAST vaccines) and days: 13, 15, 20 and 22 (for the PCV vaccines). The control group was immunized with PBS 1X at the same schedule and injection volumes as the vaccine groups. All tumor challenges, immunizations and tumor measurements were performed in the morning. Tumor growth was monitored and the volume was calculated as: tumor volume = (length × width^2^)/2. Animals were euthanized with carbon dioxide overdose (CO_2_) followed by confirmation with cervical dislocation when tumor volume reached 2000 mm^3^ or when animals became moribund with severe weight loss or ulceration. Values were reported as means ± S.E., with statistical analysis performed using Prism 8.0 (GraphPad Software). All statistical analyses were performed in: 8/10 mice in mock group, since 2 animals did not develop tumor as described before by [19]; 10/10 mice in all other treated groups.

### FS peptide array and vaccine peptide selection

To identify FSP for both vaccine approaches, we collected pre-immune sera and sera from 7-days post 4 T1 tumor challenge (*n* = 24 mice) or KPC tumor challenge (*n* = 18). A FS peptide microarray similar to our previous FS peptide microarray [10] with 788 peptides representing 200 Fs antigens as 20mer peptides was used for these studies. The FS antigens were from FS transcripts that could be generated by the errors of the RNA processing: insertion and deletion (INDEL) of the microsatellite (MS) region during the transcription and mis-splicing of the exons. The majority of these FS antigens were predicted MS INDEL FS antigens from mono-repeat MS regions (minimum repeat length was 7 nt). MS FS antigens were selected based on the length of the homopolymer and the length of the predicted FSP length. The FS antigens with longest microsatellite repeats were selected, since they have higher INDEL rates during the transcription [14] and FSPs longer than 17 amino acids were chosen. The remaining FS antigens were selected from human EST library analysis where FS with high frequencies in tumor EST libraries and low frequencies in normal EST libraries were selected [33]. Peptides were synthesized (Sigma-Aldrich, St. Louis, MO and WatsonBio, Houston, TX) without purification and spotted (Applied Microarrays, Tempe, USA) on NSB-9 amine slides (NSB Postech, Seoul, South Korea) using our previously developed methods [11]. Dilute sera was spotted on filter paper (903, Whatman) and dried overnight at room temperature. A 6 mm spot was punched and the filter paper was added to 150 μl blocking buffer (PBS 1X, 0.05% Tween-20, 3% BSA (bovine serum albumin)) with *E. coli* extract (1 mg/ml) (dilution 1:200) and probed on peptide array for 1.5 h at room temperature (R.T). Arrays were washed 3 times with PBST (PBS 1X, 0.05% Tween-20), incubated with 200 μl of 3–5 nM goat anti-mouse IgG-AlexaFluor 647 (Thermo Scientific, Waltham, MA), washed and scanned on an Innoscan 910 (Innopsys, Carbonne, France) at 80% gain at 647 nm excitation and 20% gain at 532 nm excitation. Microarray data was image-processed with GenePix Pro-6.0 (Molecular Devices, Sunnyvale, CA) and exported to Excel prior to analysis with JMP 12 (Statistical Discovery Software). Raw intensities were median normalized by slide and reactive peptides were defined as post-challenge signal two times higher than the standard deviation of the naïve mice (average). FSP positive rates were calculated for each positive peptide and the top 10 peptides with highest incidence chosen to compose the FAST vaccine. For PCVs, from the positives peptides for each mouse we selected 10 peptides with highest fluorescence intensities and that represented different frameshifts antigens. For the non-reactive FS vaccines, we selected 10 non-reactive peptides for each mouse.

### 4 T1 tumor metastasis

To evaluate the 4 T1 clonogenic spontaneous metastasis, lungs were aseptically removed at the time of sacrifice as determined by the endpoints criteria (tumor size (larger than 2000 mm^3^) and/or clinical signs of illness (lethargy, hunched posture, ulceration and etc.), ranging from 40 to 78 days post-challenge. Lungs were minced and digested with a solution of 10 mg/ml collagenase type I and 10 mg/ml hyaluronidase for 20–30 min at 37 °C under slow rotation. The suspension was filtered through 70 μm cell strainers and washed two times with complete RPMI-160 culture medium. Cells were suspended in the same medium supplemented with 60 μM 6-thioguanine (Sigma Aldrich) (10 ml/plate) and cultured in petri dishes for 14 days at 37 °C and 5% CO_2_. Plates were fixed with methanol for 5 min, carefully washed with water and stained with 0.03% methylene blue and counted. Data are expressed as total number of metastatic colonies per mouse [32].

### Peptide ELISAs

Specific IgG antibody responses in the sera of immunized mice were determined by ELISA in 96-well MaxiSorp plates (Nunc) coated with each vaccine peptide. Peptides were coated (10 μg/ml peptide/well) in carbonate-bicarbonate buffer (pH 9.6), overnight at 4 °C. Peptide-coated plates were blocked with blocking solution (PBS 1X, 0.05% Tween-20, 3% BSA) for 1.5 h a 37 °C, and washed thrice with PBS-T 1X (PBS 1X, 0.05% Tween-20). Mouse sera at the endpoint, either pooled sera by group or from individual mice, was diluted 1:200 in blocking solution, added to the plates, and incubated for 1.5 h at room temperature. Plates were washed and bound IgG was detected with horseradish peroxidase-conjugated anti-mouse IgG (Bethyl Laboratories Inc.) followed by TMB Substrate Solution (Thermo Fisher Scientific). The reaction was stopped with 0.5 M HCl and the final absorbance at 450 nm was measured in a plate reader (SpectraMax 190, Molecular devices). For the final absorbance, the pre-immune response was subtracted.

### IFN-γ ELISPOT

At endpoints, the vaccinated mice were euthanized and the spleens were aseptically removed, minced and filtered with 100 μm screens in complete RPMI culture medium (10% FBS, HEPES, L-glutamine, ß-mercatoethanol, sodium pyruvate and penicillin/streptomycin). Red blood cells were removed by lysis with BD Pharm Lyse™ (BD biosciences), splenocytes were suspended and then counted. Cells were diluted in freeze medium (complete RPMI with 10% DMSO) and stored in liquid nitrogen until use. ELISPOT plates (BD biosciences) were coated with anti-mouse IFNγ (10 μg/ml) as described by manufacturer and incubated overnight at 4 °C. Plates were washed and blocked with complete RPMI for 2 h at 37 °C with 5% CO_2_. Splenocytes were thawed and diluted to 5 × 10^6^ cells/ml in complete RPMI medium, and 100 μl cells added to each well and stimulated with FS peptide pools (3–4 FS antigens/well) (1 μg/well each FS peptide) or 4 T1 tumor cells (1 × 10^5^ cells/well). As a negative control, splenocytes were stimulated with medium only. Concanavalin A (Sigma Aldrich) was used as positive control. Plates were incubated 20–24 h for the peptide stimulation and 72–96 h for tumor cell stimulation, at 37 °C with 5% CO_2_. Plates were washed, incubated with biotinylated anti-IFN-γ according to manufacturer’s protocol, developed with AEC substrate set (BD biosciences) and spots counted by the AID EliSpot Reader System (Autoimmun Diagnostika GmbH, Germany). Final numbers are represented as sum of the spots for the all vaccine peptides.

### Flow cytometry

Frozen splenocytes prepare as described before were thawed and washed twice with complete RPMI culture medium. Cells were counted and prepared to 1 × 10^7^ cells/ml, and one million cells were re-stimulated in vitro as follows. Lymphocytes were identified by forward and side scatter, and dead cells and doublets were excluded. Cells were stained extracellularly with amine reactive viability dye (Ghost Dye™ Red 780) (TonBio Biosciences) and fluorochrome-conjugated Abs specific for: CD8a (PerCP-Cy5.5, clone 53–6.7), CD4 (PE, clone GK1.5), IFN-γ (APC, XMG1.2), Granzyme B (FITC, clone NGZB), IL-2 (PE-CF594, clone JES6-5H4), TNF-α (BV421, Clone MP6-XT22), and PD-1 (PE-Cyanine7, clone J43). Cells were surface stained ex vivo, then fixed and permeabilized for intracellular staining (Fixation/Permeabilization Solution Kit with BD GolgiStop; BD Bioscience). For the intracellular cytokine analysis, cells were stimulated for 3–4 h with: FS peptide pools (3–4 FS antigens per pool), or PMA (50 ng/ml) and ionomycin (500 ng/ml) or medium only in the presence of GolgiStop (BD Bioscience), as recommended by the manufacturer. Data were acquired on an Attune NxT Flow cytometer (Thermo Fischer Scientific) and analyzed with FlowJo™ v10.6.1 software. Gating strategy were confirmed on unstimulated control samples or fluorescence minus one controls, as appropriate. Presentation of cytokine production was performed using SPICE version 6.0 software (National Institute of Allergy and Infectious Diseases, National Institutes of Health) [34].

## Supplementary information

**Additional file 1: Supplementary Table 1.** Personal vaccines peptides selected in murine models.

**Additional file 2 Supplementary Fig. 1.** Heat map of the FS peptides selected for the NR-PCV vaccine groups. Y-axis, FS peptides; X-axis = mouse ID and time point (T0, before 4T1 challenge; T7, 7 days post challenge). Mice #ID 1 - #ID 5 were treated with NR-PCV alone; Mice #ID 11 - #ID 20 were treated with NR-PCV and ICI. **Supplementary Fig. 2.** Individual tumor growth curves for mice treated with FAST or PCVs as monotherapy. **Supplementary Fig. 3.** Tumor development of the re-challenged FAST vaccinated tumor-free mice. In the BC-FAST and PC-FAST groups, the long-term tumor free mice were re-challenged s.c. with double of number of 4T1 cells (1 x 10^3^ cells/mouse) on day 42 and they received an additional vaccine boost on day 64. No additional ICI treatment was administrated. Average 4T1 tumor growth curves without (A) and with previous ICI treatment (B). Data were analyzed by two-way ANOVA with Bonferroni multiple comparisons post-test (*** p < 0.001, ** p < 0.01). (C and D) Kaplan-Meyer tumor free curves after re-challenge. Non-statistical significance between mock group and vaccinated group were observed. **Supplementary Fig. 4.** Individual tumor growth curves for mice treated with FAST or PCVs combined with ICI. Data shown is related to Supporting Figure 3. **Supplementary Fig. 5.** Individual tumor growth curves for re-challenged mice treated with FAST or PCVs combined with ICI or alone. Data shown is related to Supporting Figure 3. **Supplementary Fig. 6.** Evaluation of the specific IgG immune response to FS candidates after vaccine regimen. 96-wells ELISA plates were coated with 0.5 μg peptide/well overnight at 4 ºC. Pooled sera (FAST groups) and individual serum (PCV groups) at the endpoint were diluted 1:200 and incubated for 1.5 h at room temperature. Absorbance at 450 nm was measured and final values obtained after subtracting pre-immune reactivity by the respective group. Sera were tested in triplicate. **Supplementary Fig. 7.** Characterization of specific T cell immune response to FAST and PCV peptide pools by IFNγ ELISPOT. Mice splenocytes were tested against 3 different peptide pools, with each pool composed by 3-4 FS peptides. Each vaccine peptide pool was prepared specifically for the FAST formulations and for mouse-matched PCVs. (A) BC-FAST; (B) BC-PCV; (C) BC-FAST + ICI; (D) BC-PCV + ICI; (E) PC-FAST (F) PC-FAST + ICI; (G) NR-PCV + ICI. (A, B, C, E and F) lines represent mock group baseline immune response to the peptide pools (black= peptide pool 1; red= peptide pool 2; blue= peptide pool 3). Due to the limited number of cells, mock group splenocytes were tested against each PCV not in pools but as one (black “X”). Data shown is related to Fig. 5.

## Data Availability

The datasets used and/or analyzed during the current study are available from the corresponding author on reasonable request.

## Abbreviations

FSP: Frameshift peptides
FAST vaccine: Frameshift Antigen Shared Therapeutic vaccine
PCV: Personal cancer vaccine
ICI: Immune Checkpoint inhibitor
SNV: Single nucleotide variants
FS: Frameshift neoantigens
FBS: Fetal bovine serum
PBS: Phosphate buffered saline
S.c: Subcutaneously
Poly (I:C): Polyinosinic–polycytidylic acid potassium salt
INDEL: Insertion and deletion
MS: Microsatellite
BSA: Bovine serum albumin
R.T.: Room temperature
PBS-T: PBS 1X, 0.05% Tween-20
BC-PCV: Breast cancer personalized vaccine
BC-FAST: Breast cancer FAST vaccine
PC-FAST: Pancreatic cancer FAST vaccine
NR: Non-reactive vaccine.
