# Supplementary material for: Comparison of personal and shared frameshift neoantigen vaccines in a mouse mammary cancer model
Source: BMC Immunol. 2020 May 5;21:25. doi: 10.1186/s12865-020-00350-3 (PMC7201681; doi:10.1186/s12865-020-00350-3)
Supplement: Supplementary file 2 — Additional file 2 Supplementary Fig. 1. Heat map of the FS peptides selected for the NR-PCV vaccine groups. Y-axis, FS peptides; X-axis = mouse ID and time point (T0, before 4T1 challenge; T7, 7 days post challenge). Mice #ID 1 - #ID 5 were treated with NR-PCV alone; Mice #ID 11 - #ID 20 were treated with NR-PCV and ICI. Supplementary Fig. 2. Individual tumor growth curves for mice treated with FAST or PCVs as monotherapy. Supplementary Fig. 3. Tumor development of the re-challenged FAST vaccinated tumor-free mice. In the BC-FAST and PC-FAST groups, the long-term tumor free mice were re-challenged s.c. with double of number of 4T1 cells (1 x 10^3^ cells/mouse) on day 42 and they received an additional vaccine boost on day 64. No additional ICI treatment was administrated. Average 4T1 tumor growth curves without (A) and with previous ICI treatment (B). Data were analyzed by two-way ANOVA with Bonferroni multiple comparisons post-test (*** p < 0.001, ** p < 0.01). (C and D) Kaplan-Meyer tumor free curves after re-challenge. Non-statistical significance between mock group and vaccinated group were observed. Supplementary Fig. 4. Individual tumor growth curves for mice treated with FAST or PCVs combined with ICI. Data shown is related to Supporting Figure 3. Supplementary Fig. 5. Individual tumor growth curves for re-challenged mice treated with FAST or PCVs combined with ICI or alone. Data shown is related to Supporting Figure 3. Supplementary Fig. 6. Evaluation of the specific IgG immune response to FS candidates after vaccine regimen. 96-wells ELISA plates were coated with 0.5 μg peptide/well overnight at 4 ºC. Pooled sera (FAST groups) and individual serum (PCV groups) at the endpoint were diluted 1:200 and incubated for 1.5 h at room temperature. Absorbance at 450 nm was measured and final values obtained after subtracting pre-immune reactivity by the respective group. Sera were tested in triplicate. Supplementary Fig. 7. Characterization of specific T cell immune re [file 12865_2020_350_MOESM2_ESM.docx]

Supporting Information for:

**Comparison of Personal and Shared Frameshift Neoantigen Vaccines in a Mouse Mammary Cancer Model**

Milene Peterson^1^, Sierra Nicole Murphy^1^, John Lainson ^1^, Jian Zhang^1^, Luhui Shen^1^, Chris W. Diehnelt ^1^, Stephen Albert Johnston^1,2^.

^1^Center for Innovations in Medicine, The Biodesign Institute, Arizona State University, Tempe, Arizona; ^2^Calviri, Inc, Tempe, AZ

SUPPLEMENTARY INFORMATION

**
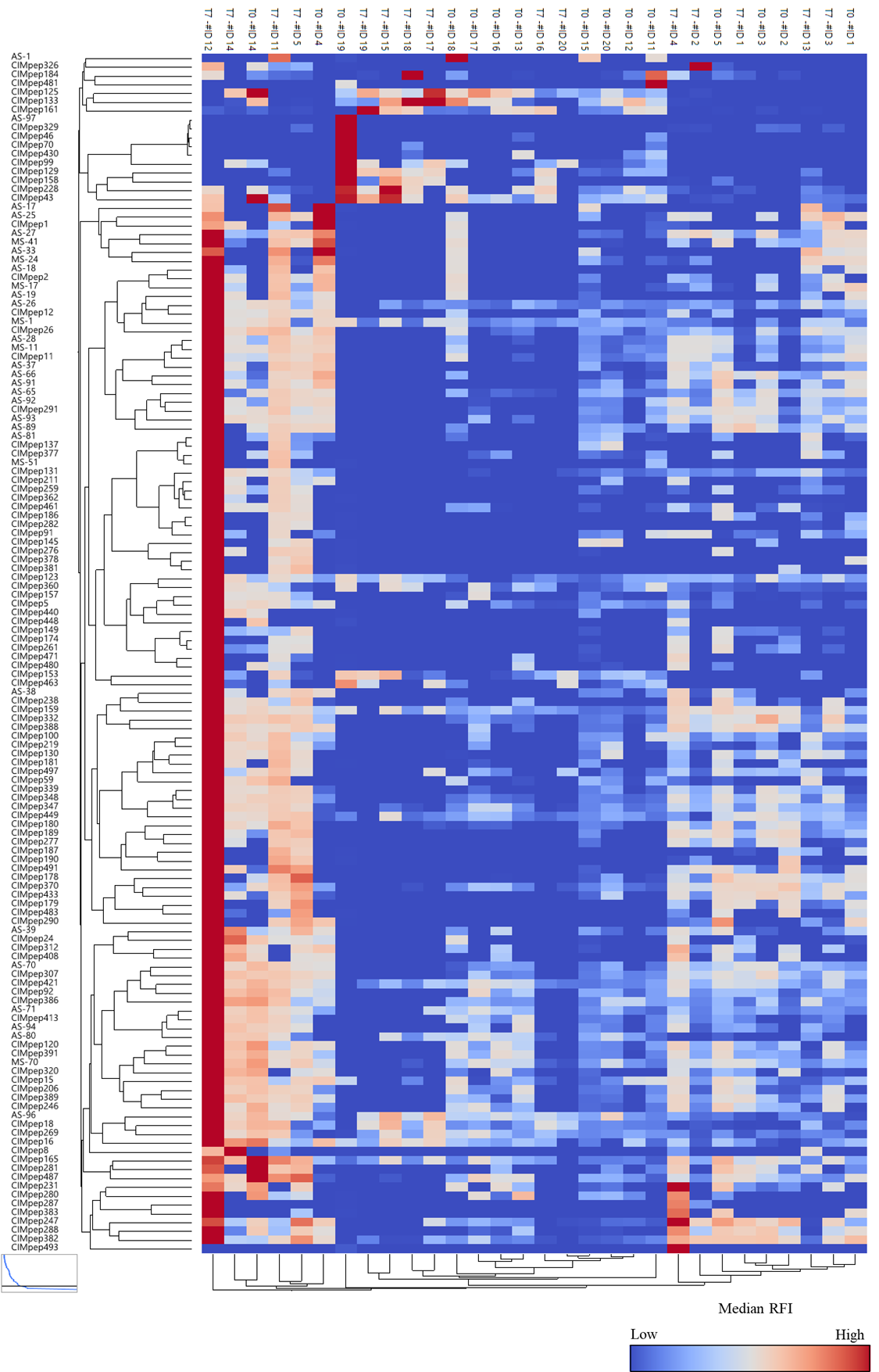
**

**Supplementary Fig. 1.** Heat map of the FS peptides selected for the NR-PCV vaccine groups. Y-axis, FS peptides; X-axis = mouse ID and time point (T0, before 4T1 challenge; T7, 7 days post challenge). Mice #ID 1 - #ID 5 were treated with NR-PCV alone; Mice #ID 11 - #ID 20 were treated with NR-PCV and ICI.


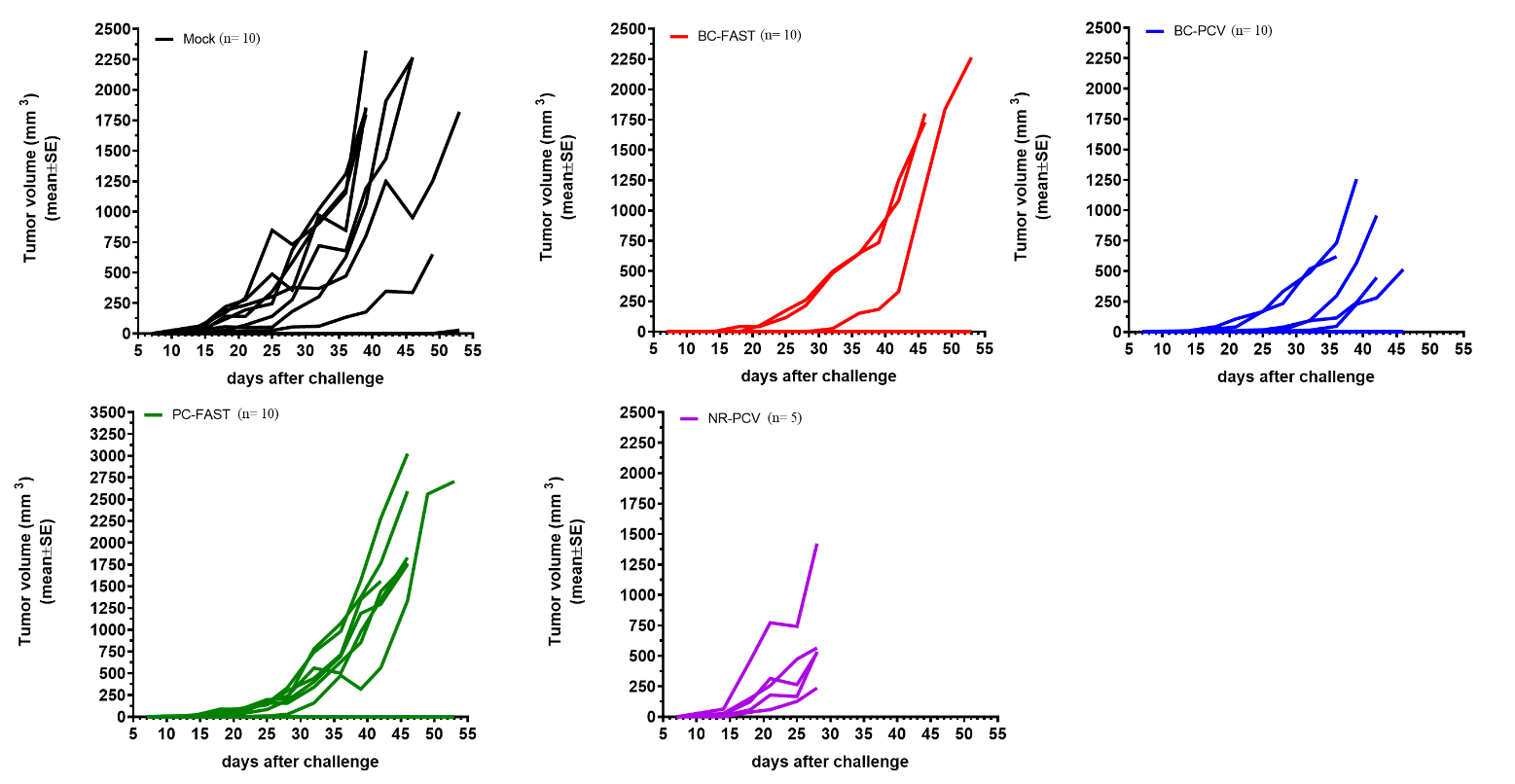


**Supplementary Fig. 2.** Individual tumor growth curves for mice treated with FAST or PCVs as monotherapy.

**a**

**b**


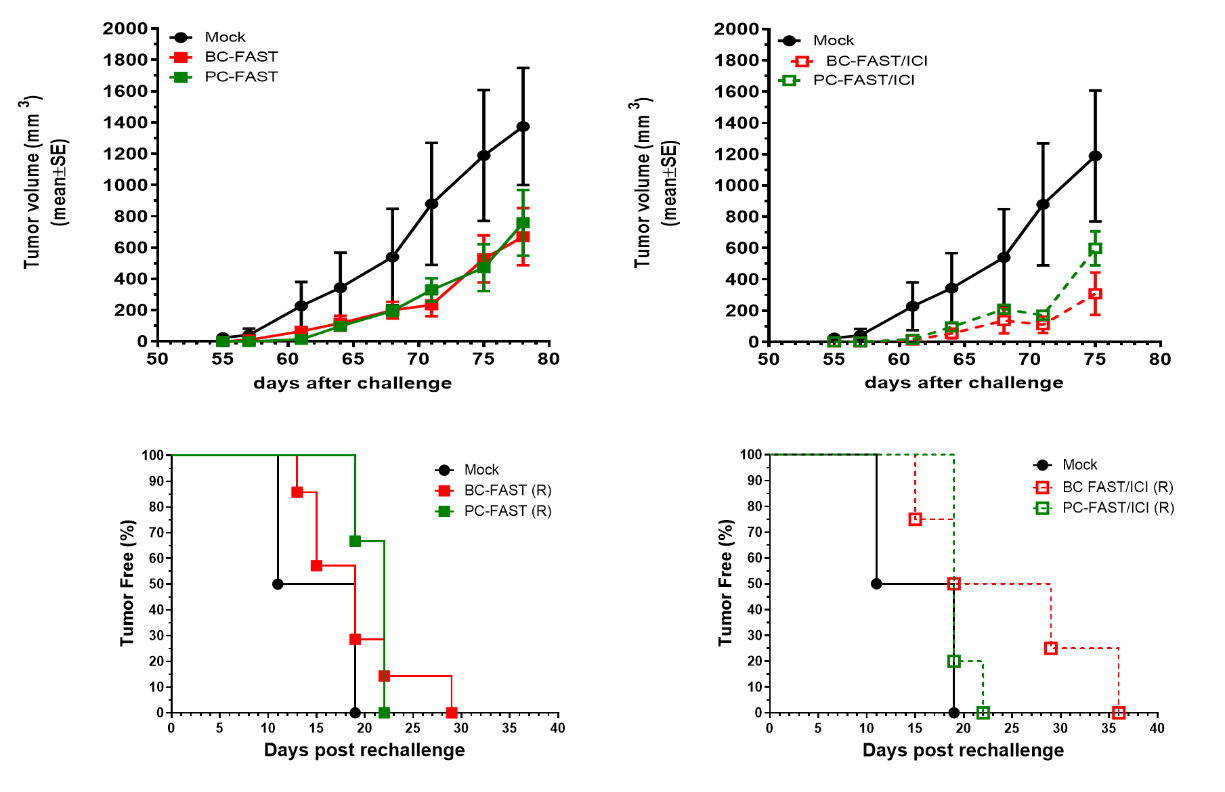


**c**

**d**

**Supplementary Fig. 3.** Tumor development of the re-challenged FAST vaccinated tumor-free mice. In the BC-FAST and PC-FAST groups, the long-term tumor free mice were re-challenged s.c. with double of number of 4T1 cells (1 x 10^3^ cells/mouse) on day 42 and they received an additional vaccine boost on day 64. No additional ICI treatment was administrated. Average 4T1 tumor growth curves without (A) and with previous ICI treatment (B). Data were analyzed by two-way ANOVA with Bonferroni multiple comparisons post-test (*** p < 0.001, ** p < 0.01). (C and D) Kaplan-Meyer tumor free curves after re-challenge. Non-statistical significance between mock group and vaccinated group were observed.


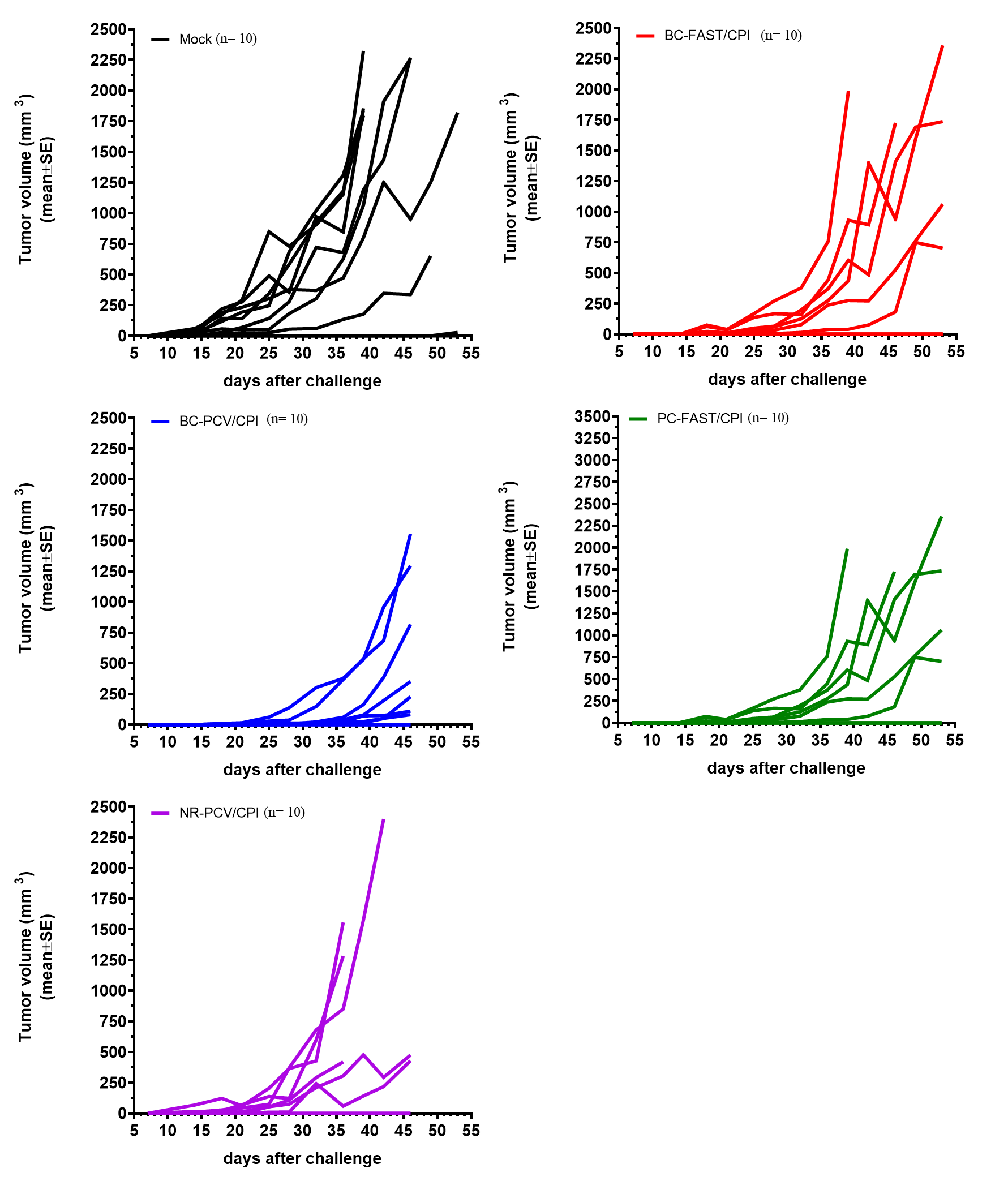


**Supplementary Fig. 4.** Individual tumor growth curves for mice treated with FAST or PCVs combined with ICI. Data shown is related to Supporting Figure 3.


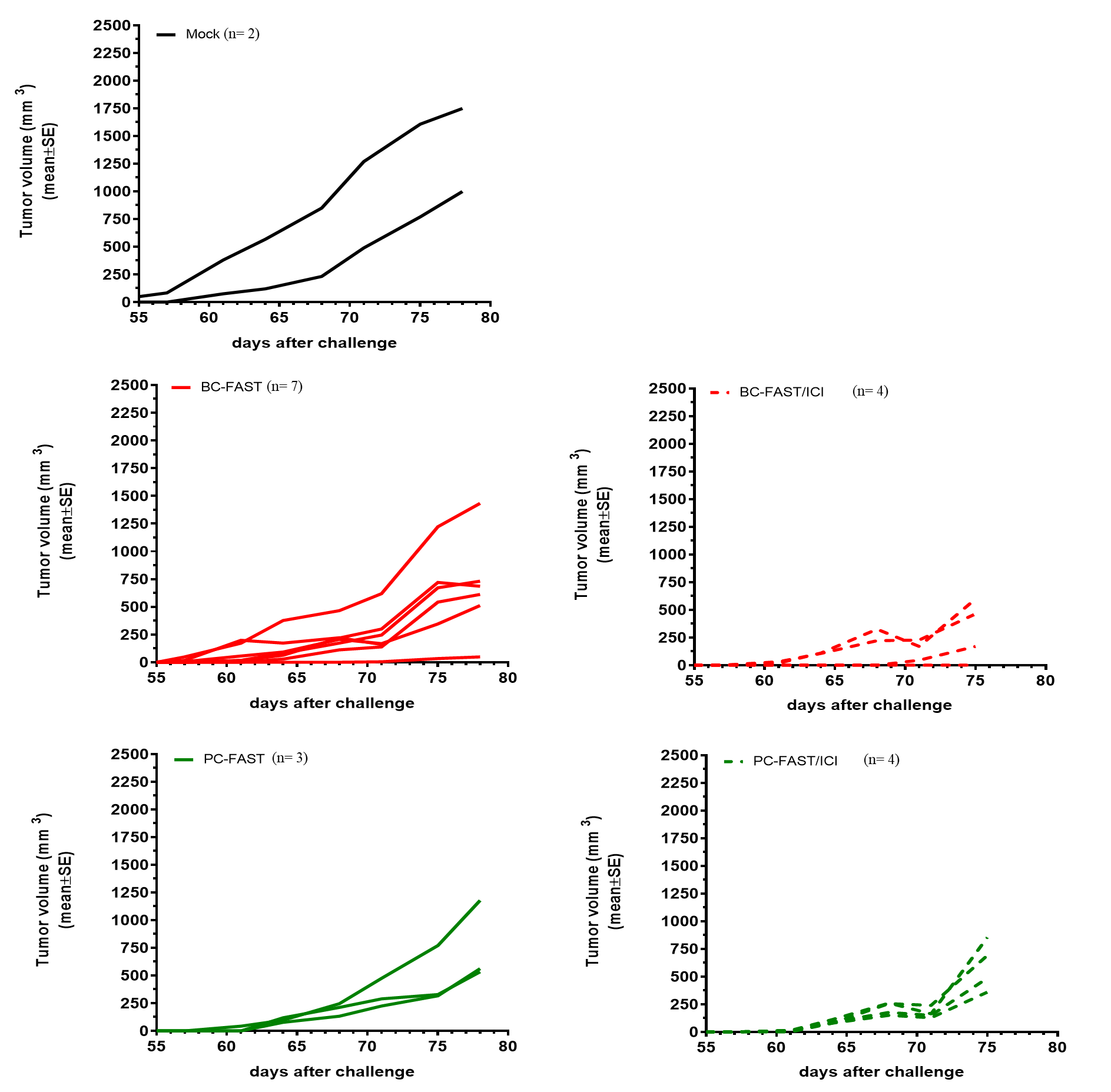


**Supplementary Fig. 5.** Individual tumor growth curves for re-challenged mice treated with FAST or PCVs combined with ICI or alone. Data shown is related to Supporting Figure 3.

**
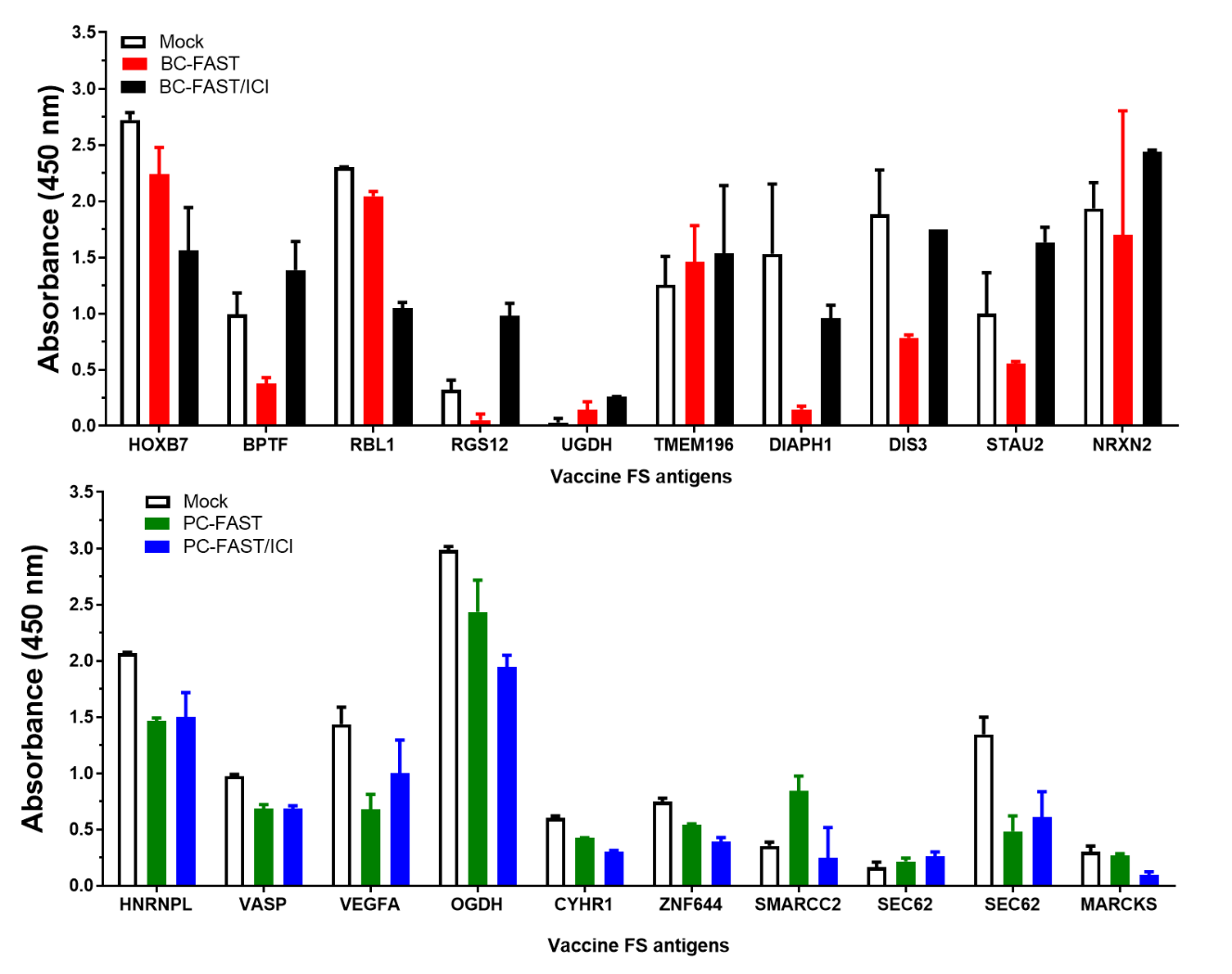
****Supplementary Fig. 6. Evaluation of the specific IgG immune response to FS candidates after vaccine regimen.** 96-wells ELISA plates were coated with 0.5 µg peptide/well overnight at 4 ºC. Pooled sera (FAST groups) and individual serum (PCV groups) at the endpoint were diluted 1:200 and incubated for 1.5 h at room temperature. Absorbance at 450 nm was measured and final values obtained after subtracting pre-immune reactivity by the respective group. Sera were tested in triplicate.

**c**

**b**

**a**

PC-FAST/ICI

PC-FAST

BC-PCV

BC-FAST

BC-PCV/ICI

BC-FAST/ICI

NR-PCV/ICI

**Supplementary Fig. 7.** **Characterization of specific T cell immune response to FAST and PCV peptide pools by IFNγ ELISPOT.** Mice splenocytes were tested against 3 different peptide pools, with each pool composed by 3-4 FS peptides. Each vaccine peptide pool was prepared specifically for the FAST formulations and for mouse-matched PCVs. (A) BC-FAST; (B) BC-PCV; (C) BC-FAST + ICI; (D) BC-PCV + ICI; (E) PC-FAST (F) PC-FAST + ICI; (G) NR-PCV + ICI. (A, B, C, E and F) lines represent mock group baseline immune response to the peptide pools (black= peptide pool 1; red= peptide pool 2; blue= peptide pool 3). Due to the limited number of cells, mock group splenocytes were tested against each PCV not in pools but as one (black “X”). Data shown is related to Fig. 5.
